# Villanova ReachOut Forming Connections with Older Adults

**DOI:** 10.1093/geroni/igab046.2754

**Published:** 2021-12-17

**Authors:** Christina Whitehouse, Catherine Curley, Caitlin Gomes, Michelle McKay, Christine Brewer, Melissa O'Connor

**Affiliations:** 1 Villanova University, Villanova Univesity, Pennsylvania, United States; 2 Villanova University, Villanova University, Pennsylvania, United States; 3 Villanova University, M. Louise Fitzpatrick College of Nursing, Villanova, Pennsylvania, United States

## Abstract

Older adults are at increased risk for loneliness and social isolation. Research on loneliness identifies increased rates of depression, increased cognitive decline, and poor cardiovascular health outcomes. The COVID-19 pandemic forced many older adults into social isolation for protection against this insidious virus. Mandated lockdowns and personal decisions to shelter-in-place produced a tremendous increase in rates of loneliness, especially among older adults. Identifying a need for communication and relationships, we created Villanova ReachOut, a program that partners interprofessional students (N= 66) with older adults (N=53) through weekly telephone or video calls. To assess the impact of our program we developed a five question survey administered via phone to older adults and a 13-item survey for volunteers to assess training, satisfaction, needs and impact of the program. Of the older adults (n=16) who completed the survey, 78.6% believe the program helped them feel less isolated throughout the pandemic and 93.8% indicated they looked forward to weekly calls with their partner . Volunteers who completed the survey (N=25), overwhelmingly stated they enjoy and look forward to their calls (100%) and their communication skills have improved (92%). Volunteers reported being paired up with an older adult for weekly conversation had a positive impact on their personal and professional development. Findings from our program evaluation provide rich data in descriptions of positive impact for both the older adult and volunteer. These findings also support the need for programs that engage in intergenerational dialogue, specifically targeting older adults and the potential older adult workforce.

